# Clinical features as potential prognostic factors in patients treated with nivolumab for highly pretreated metastatic gastric cancer: a multicenter retrospective study

**DOI:** 10.1186/s12885-021-09118-3

**Published:** 2022-01-03

**Authors:** Akihiko Sano, Makoto Sohda, Nobuhiro Nakazawa, Yasunari Ubukata, Kengo Kuriyama, Akiharu Kimura, Norimichi Kogure, Hisashi Hosaka, Atsushi Naganuma, Masanori Sekiguchi, Kana Saito, Kyoichi Ogata, Makoto Sakai, Hiroomi Ogawa, Ken Shirabe, Hiroshi Saeki

**Affiliations:** 1 Department of General Surgical Science, Gunma University Graduate School of Medicine, 3-39-22 Showa-machi, Maebashi, Gunma 371-8511 Japan; 2 Department of Gastroenterology, Gunma Prefectural Cancer Center, 617-1 Takabayashi nishi-machi, Ohta, Gunma 373-8550 Japan; 3 Department of Gastroenterology, National Hospital Organization Takasaki General Medical Center, 36 Takamatsu-machi, Takasaki, Gunma 370-0829 Japan; 4 Department of Gastroenterology, Isesaki Municipal Hospital, 12-1 Tsunatori hon-machi, Isesaki, Gunma 372-0817 Japan; 5 Department of Surgery, Japan Community Healthcare Organization Gunma Central Hospital, 1-7-13 Kouuncho, Maebashi, Gunma 371-0025 Japan

**Keywords:** Nivolumab, Advanced gastric cancer, Performance status, Trastuzumab, Immune-related adverse events

## Abstract

**Background:**

Although nivolumab (anti-programmed cell death-1 antibody) is a promising approach for advanced gastric cancer (AGC), the response rate remains limited. The aim of this multicenter retrospective study was to determine if clinical features could serve as prognostic factors of the efficacy of nivolumab in patients with AGC.

**Methods:**

Fifty-eight patients with AGC who were treated with nivolumab as a third or later line from October 2017 to December 2018 at any of five clinical sites were enrolled in the study. The correlation between the best overall response and clinical features was investigated. Overall survival and progression-free survival after initiation of nivolumab were calculated and clinical features that could be predictors of the prognosis were sought.

**Results:**

The disease control rate (DCR) for nivolumab was 36.2% and was significantly correlated with performance status (*p* = 0.021), metastasis to one organ (*p* = 0.006), and grade 2 or higher immune-related adverse events (*p* = 0.027). There was also a significant association between response to nivolumab and ability to receive subsequent chemotherapy (*p* = 0.022). In the analysis of overall survival, the following variables were identified as being significantly associated with a poor outcome: Eastern Cooperative Oncology Group performance status ≥1, prior treatment with trastuzumab, no immune-related adverse events, lack of a response to nivolumab, and inability to receive subsequent chemotherapy.

**Conclusion:**

The findings of this study suggest that nivolumab may be ineffective for AGC in patients with poor performance status and those with a history of treatment with trastuzumab.

## Background

Despite the recent advent of various anticancer drugs, there is still no cure for unresectable advanced or recurrent gastric cancer (AGC). According to the Japanese gastric cancer treatment guidelines [1], oral fluoropyrimidine plus platinum is the standard first-line chemotherapy for human epidermal growth factor receptor 2 (HER2)-negative unresectable AGC [2–6]. In contrast, trastuzumab is recommended in combination with first-line chemotherapy in patients with HER-2-positive AGC based on the results of the ToGA trial [7]. For second-line chemotherapy, paclitaxel plus ramucirumab, an anti-vascular endothelial growth factor receptor 2 antibody, was shown to be superior to weekly paclitaxel monotherapy in a RAINBOW trial [8]. In a large Phase III ATTRACTION-2 study, nivolumab, a monoclonal antibody targeting programmed cell death-1 (PD-1), was shown to have significant survival benefits compared with placebo in patients with advanced gastric or esophagogastric junction cancer [9]. After the results of this study were published, nivolumab monotherapy was recommended as a third-line treatment for patients with unresectable advanced or recurrent gastric/esophagogastric junctional cancer in Japan. Furthermore, the long-term efficacy of nivolumab monotherapy was confirmed at the 3-year follow-up [10]. In this study, median overall survival (OS) was significantly longer in the nivolumab monotherapy group than in the placebo group (5.3 months vs 4.1 months; 3-year survival rate, 5.6% vs 1.9%; hazard ratio (HR) = 0.62, *p* < 0.0001). And a survival benefit of treatment beyond progression with nivolumab was suggested. Although this anti-PD-1 monoclonal antibody is a promising approach for patients with advanced gastric cancer, the response rate is still limited. There is a need to identify novel biomarkers that could help identify patients who would benefit from nivolumab and those with primary resistance.

In this multicenter retrospective study, we analyzed the clinical features of patients with unresectable AGC who received nivolumab to identify if any of these features could serve as potential prognostic markers.

## Methods

### Patients and data collection

Patients with AGC that was histologically confirmed to be adenocarcinoma who were treated with nivolumab monotherapy as third-line or later line between October 2017 and December 2018 at Gunma University Hospital, Gunma Prefectural Cancer Center, National Hospital Organization Takasaki General Medical Center, Isesaki Municipal Hospital, or Japan Community Healthcare Organization Gunma Central Hospital were retrospectively reviewed. Patients who had previously received immunotherapy were excluded. The following clinical data on patient characteristics were retrospectively collected from the medical records: age, sex, Eastern Cooperative Oncology Group performance status (ECOG PS), disease status (metastatic or relapsed), primary site, histological type (Lauren classification), HER-2 status, site of metastasis, organs with metastasis, previous treatment regimens, and therapies before initiating treatment with nivolumab.

### Treatment and assessment

Nivolumab was administered intravenously at a dose of 3 mg/kg or 240 mg flat dose every 2 weeks until disease progression, clinical deterioration, unacceptable toxicity occurred, or the patient refused to continue treatment. The best overall response was evaluated and classified as complete remission (CR), partial response (PR), stable disease (SD), or progressive disease (PD) according to the Response Evaluation Criteria in Solid Tumors (RECIST) guidelines version 1.1 [11] using computed tomography at 6–8-week intervals during nivolumab therapy. Patients with a SD, PR, or CR were considered to be “responders” and those with PD were assumed to be “non-responders”. With regard to the safety analysis, adverse events (AEs) linked to use of nivolumab were evaluated according to the National Cancer Institute Common Terminology Criteria for Adverse Events version 5.0 and included immune-related AEs (irAEs). In previous studies [12–14], irAEs were defined as AEs with a potential immune cause, events for which frequent monitoring was needed, or for which immunosuppressive and/or endocrine therapy was prescribed according to severity. OS and progression-free survival (PFS) were assessed from the date of initiation of treatment with nivolumab. OS was measured until death or censored at the latest follow-up for surviving patients. PFS was measured until progression or death from any cause and censored at the date when the patient was last confirmed to be progression-free.

### Statistical analysis

Differences between two groups were compared using Fisher’s exact test for categorical variables and the Mann-Whitney *U* test for quantitative variables. Survival curves were constructed using the Kaplan-Meier method and compared using the log-rank test. The Cox proportional hazards regression model was used to calculate HRs with 95% confidence intervals (CIs). All data were analyzed using EZR, which is a freely available easy-to-use medical statistical software package [15]. A *p*-value < 0.05 was considered statistically significant.

## Results

### Clinical characteristics of patients with AGC treated by nivolumab

The study population consisted of 58 patients who were treated with nivolumab for AGC. Clinical characteristics of patients in this study were listed in Table 1. The 58 patients included 45 men (78%) and 13 women (22%). The median age at the time of initiation of nivolumab was 66 years (range, 38–82). Eight patients (14%) had an ECOG PS of 0 and 50 (86%) had an ECOG PS of ≥1. At diagnosis, 43 patients (74%) were classified as metastatic and 15 (26%) as relapsed. Forty-nine patients (84%) had gastric cancer and nine (16%) had esophagogastric junction cancer. Thirty-four patients (59%) had intestinal type and 13 (22%) had HER-2 positive disease. Seventeen patients (29%) had metastasis to one organ, and 41 (71%) had metastasis to two or more organs. Fifty-six patients (97%) received regimens containing pyrimidine analogs, 50 (86%) received platinum-containing regimens, 55 (95%) received a taxane, and 48 (83%) received regimens containing ramucirumab. In 58 patients treated with nivolumab, none of the patients achieved a CR. Four patients achieved a PR (7%), 17 achieved SD (29%), and the remaining 37 had PD (64%), resulting in an objective response rate of 7% and a disease control rate (DCR) of 36% (Table 2). In this study population, no obvious Pseudo-progression or hyper-progression was observed. Table 3 summarizes the clinical characteristics for patients treated with nivolumab in responder and non-responder groups. Most of the clinical characteristics were similarly distributed between the patients who responded to nivolumab and those who did not. However, the DCR was significantly correlated with ECOG PS of 0 (*p* = 0.021) and with metastasis to one organ (*p* = 0.006).

**Table 1:** Characteristics of patients before treatment of nivolumab

Variables	*n* = 58
	No. (%)
Sex	
Male	45 (78%)
Female	13 (22%)
Age (years) (Median, Range)	66 (38–82)
ECOG performance status	
0	8 (14%)
1	36 (62%)
2	12 (21%)
3	2 (3%)
Disease status	
Metastatic	43 (74%)
Relapsed	15 (26%)
Primary site	
Gastric	49 (84%)
Esophagogastric junction	9 (16%)
Histological type	
Intestinal type	34 (59%)
Diffuse type	24 (41%)
HER-2 status	
Positive	13 (22%)
Site of metastases	
Lymph nodes	41 (72%)
Peritoneum	27 (47%)
Liver	20 (34%)
Lung	12 (21%)
Bone	9 (16%)
Other	7 (12%)
Organs with metastasis	
1	17 (29%)
2	24 (42%)
3	13 (22%)
≥ 4	4 (7%)
Previous treatment regimens	
2	35 (60%)
3	18 (31%)
≥ 4	5 (9%)
Previous therapies	
Pyrimidine analogs	56 (97%)
Platinum	50 (86%)
Taxane	55 (95%)
Ramucirumab	48 (83%)
Trastuzumab	11 (19%)
Irinotecan	16 (28%)
Treatment period before nivolumab (months)	14.9 (4.9–64.6)
Immune-related adverse events	
Positive	10 (17%)

**Table 2:** Best overall responses to nivolumab (*n* = 58)

Best overall response	n (%)
Complete remission (CR)	0
Partial response (PR)	4 (7%)
Stable disease (SD)	17 (29%)
Progressive disease (PD)	37 (64%)
Objective response rate (ORR, CR + PR)	4 (7%)
Disease control rate (DCR, ORR + SD)	21 (36%)

**Table 3:** Characteristics of patients treated with nivolumab in responder and non-responder groups

Variables	Responder (*n* = 21)	Non-responder (*n* = 37)	*p*
	No. (%)	No. (%)	
Sex			
Male	18 (86%)	27 (73%)	0.338
Female	3 (14%)	10 (27%)	
Age (years) (Median, Range)	66 (53–76)	66 (38–82)	0.581
ECOG performance status			
0	6 (29%)	2 (5%)	0.021
≥ 1	15 (71%)	35 (95%)	
Disease status			
Metastatic	16 (76%)	27 (73%)	1
Relapsed	5 (24%)	10 (27%)	
Primary site			
Gastric	17 (81%)	32 (87%)	0.71
Esophagogastric junction	4 (19%)	5 (14%)	
Histological type			
Intestinal type	12 (57%)	22 (60%)	1
Diffuse type	9 (43%)	15 (40%)	
HER-2 status			
Positive	2 (10%)	11 (30%)	0.106
Site of metastases			
Lymph nodes	13 (62%)	28 (76%)	0.369
Hematogenous	9 (43%)	24 (65%)	0.167
Peritoneum	12 (57%)	15 (41%)	0.278
Organs with metastasis			
1	11 (52%)	6 (16%)	0.006
≥ 2	10 (48%)	31 (84%)	
Previous treatment regimens			
2	14 (67%)	21 (57%)	0.704
3	5 (24%)	13 (35%)	
≥ 4	2 (9%)	3 (8%)	
Previous therapies			
Pyrimidine analogs	20 (95%)	36 (97%)	1
Platinum	18 (86%)	32 (87%)	1
Taxane	20 (95%)	35 (95%)	1
Ramucirumab	16 (76%)	32 (87%)	0.471
Trastuzumab	2 (10%)	9 (24%)	0.296
Irinotecan	5 (24%)	11 (30%)	0.764
Treatment period before nivolumab (months)	17.3 (8.1–37.1)	14.6 (4.9–64.6)	0.225
Immune-related adverse events			
Positive	7 (33%)	3 (8%)	0.027

### Safety of nivolumab monotherapy

Treatment-related adverse events (TRAEs) are summarized in Table 4. Thirty-one patients (53%) experienced TRAEs; these were grade 2 or higher in 18 patients (31%). The most common TRAEs were anorexia (*n* = 9), malaise (*n* = 6), and nausea (*n* = 3). Grade 2 or higher TRAEs were observed in 10 patients (17%), with anorexia in 3 (5%), and upper gastrointestinal hemorrhage in 2 (3%). Ten patients (17%) experienced grade 2 or higher immune-related adverse events (irAEs); liver enzyme elevation (*n* = 3), and peripheral sensory neuropathy and hypothyroidism occurred in 2 patients each. The DCR was significantly higher in patients with grade 2 or higher irAEs (*p* = 0.027; Table 3).

**Table 4:** Treatment-related adverse events (TRAEs)

Treatment-related adverse events	n (%)	
	Any grade	≥grade 2
All TRAEs	31 (53%)	18 (31%)
Common TRAEs	25 (43%)	10 (17%)
Anorexia	9 (16%)	3 (5%)
Malaise	6 (10%)	0
Nausea	3 (5%)	0
Upper gastrointestinal hemorrhage	2 (3%)	2 (3%)
Fever	2 (3%)	0
Localized edema	2 (3%)	0
Creatinine increased	2 (3%)	0
Dysgeusia	2 (3%)	0
Pruritus	2 (3%)	0
Nail change	2 (3%)	0
Anemia	1 (2%)	1 (2%)
Palpitations	1 (2%)	1 (2%)
Colonic perforation	1 (2%)	1 (2%)
Neutrophil count decreased	1 (2%)	1 (2%)
Dyspnea	1 (2%)	1 (2%)
Pleural effusion	1 (2%)	1 (2%)
Eosinophilia	1 (2%)	0
Constipation	1 (2%)	0
Platelet count decreased	1 (2%)	0
Proteinuria	1 (2%)	0
Cough	1 (2%)	0
Immune-related adverse events (irAEs)	20 (35%)	10 (17%)
Liver enzyme elevation	7 (12%)	3 (5%)
Peripheral sensory neuropathy	4 (7%)	2 (3%)
Rash maculopapular	4 (7%)	1 (2%)
Colitis	4 (7%)	0
Hypothyroidism	2 (3%)	2 (3%)
Hyperglycemia	1 (2%)	1 (2%)
Esophagitis	1 (2%)	1 (2%)
Hypopituitarism	1 (2%)	1 (2%)

### Survival in responders and non-responders

**Fig. 1:**
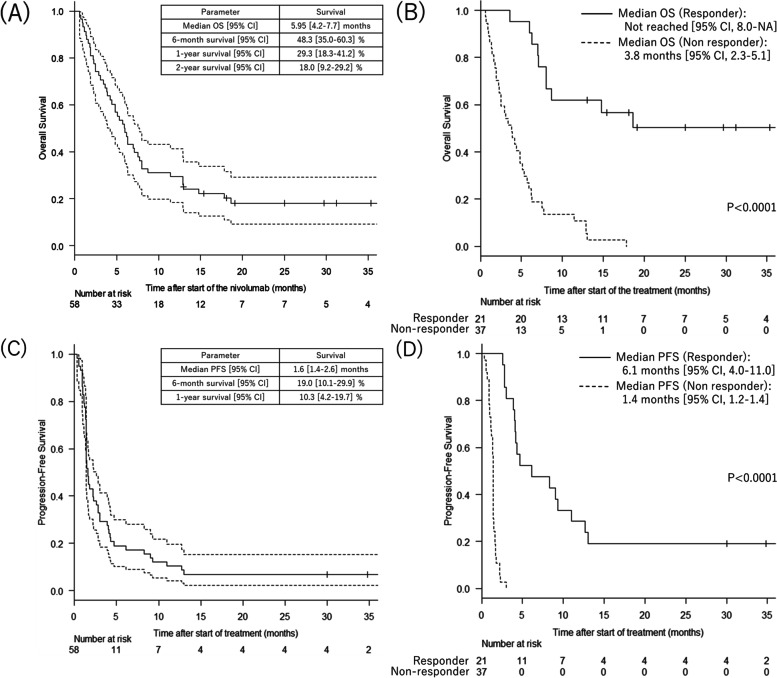
Overall (**A, B**) and progression-free (**C, D**) Kaplan-Meier survival curves for patients treated with nivolumab. After start of the nivolumab therapy, median overall survival (**A**) was 5.95 months and median progression-free survival (**C**) was 1.6 months. Patients who responded to nivolumab had significantly better overall survival (**B**) and progression-free survival (**D**) (*p* < 0.0001)

The Kaplan-Meier curves for OS and PFS are shown in Fig. 1. The median OS was 5.95 months (95% CI 4.2–7.7) and median PFS was 1.6 months (95% CI 1.4–2.6); Fig. 1A, C). Both OS and PFS were significantly longer in responders than in non-responders (Fig. 1B, D). Median OS was not reached (95% CI 8.0–NA) in responders and was 3.8 months (95% CI 2.3–5.1) in non-responders (*p* < 0.0001).

### Association of nivolumab response with subsequent chemotherapy

**Fig. 2:**
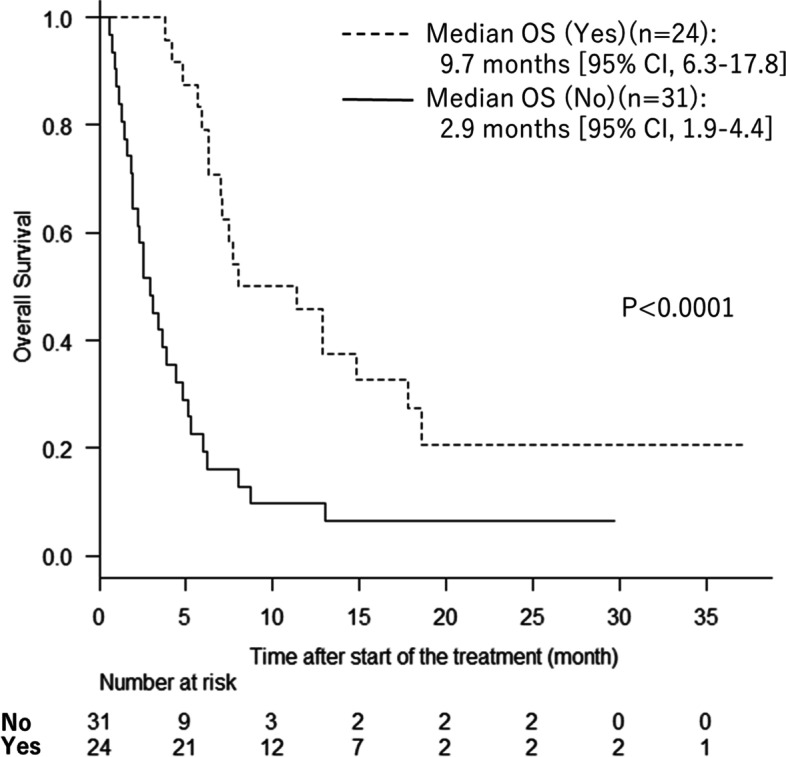
Patients who were able to receive therapy subsequent to nivolumab showed significantly better overall survival than those who were not (*p* < 0.0001)

The association between response to nivolumab and subsequent chemotherapy after nivolumab is shown in Table 5. At the time of analysis, three of the 58 patients were continuing to receive nivolumab. Chemotherapy was able to be started in 24 (44%) of 55 patients who were finally judged to have PD but not in 31 (56%). The nivolumab responder group had a significantly higher rate of subsequent chemotherapy than the non-responder group (*p* = 0.022). Fifteen patients (62%) received irinotecan subsequent to nivolumab, 9 (38%) received a taxane, 7 (29%) received a pyrimidine analog, 6 (25%) received platinum, 6 (25%) received ramucirumab, and 2 (8%) received trifluridine/tipiracil. The characteristics of the 55 patients in whom subsequent chemotherapy was or was not possible are shown in Table 6. The ability to receive subsequent chemotherapy was significantly correlated with ECOG PS at the start of nivolumab therapy (*p* < 0.001) and whether or not there was a prior trastuzumab use (*p* = 0.015). There was a significant correlation between responsiveness to nivolumab and being able to undergo chemotherapy subsequent to this agent (*p* = 0.022). Figure 2 shows the Kaplan-Meier curves for OS in patients in whom subsequent chemotherapy was possible and those in whom it was not. Median OS was 9.7 months (95% CI 6.3–17.8) in the group that subsequently received chemotherapy and 2.9 months (95% CI 1.9–4.4) in the group that did not (*p* < 0.0001).

**Table 5:** Subsequent chemotherapy after nivolumab (*n* = 55)

Subsequent chemotherapy	All patients (*n* = 55)	Responder (*n* = 18)	Non-responder (*n* = 37)	*p*
No	31 (56%)	6 (33%)	25 (68%)	0.022
Yes	24 (44%)	12 (67%)	12 (32%)	
Treatment after nivolumab	All patients (*n* = 24)	Responder (*n* = 12)	Non-responder (*n* = 12)	*p*
Irinotecan	15 (62%)	6 (50%)	9 (75%)	0.226
Taxane	9 (38%)	6 (50%)	3 (25%)	0.411
Pyrimidine analog	7 (29%)	3 (25%)	4 (33%)	0.673
Platinum	6 (25%)	3 (25%)	3 (25%)	1
Ramucirumab	6 (25%)	4 (33%)	2 (17%)	0.645
Trifluridine/tipiracil	2 (8%)	2 (17%)	0 (0%)	0.411

**Table 6:** Characteristics of patients with or without subsequent chemotherapy after treatment of nivolumab

Variables	All patients (*n* = 55)	Subsequent chemotherapy		*p*
	No. (%)	Yes (*n* = 24)	No (*n* = 31)	
Sex				
Male	42 (76%)	18 (75%)	24 (77%)	1
Female	13 (23%)	6 (25%)	7 (23%)	
Age (years) (Median, Range)	66 (38–82)	66 (38–82)	68 (49–80)	0.48
ECOG performance status				
0	8 (14%)	8 (33%)	0 (0%)	< 0.001
≥ 1	47 (86%)	16 (67%)	31 (100%)	
Disease status				
Metastatic	41 (74%)	18 (75%)	23 (74%)	1
Relapsed	14 (26%)	6 (25%)	8 (26%)	
Primary site				
Gastric	48 (87%)	22 (92%)	26 (84%)	0.451
Esophagogastric junction	7 (13%)	2 (8%)	5 (16%)	
Histological type				
Intestinal type	31 (56%)	12 (50%)	19 (61%)	0.426
Diffuse type	24 (44%)	12 (50%)	12 (39%)	
HER-2 status				
Positive	13 (24%)	3 (12%)	10 (32%)	0.116
Site of metastases				
Lymph nodes	38 (69%)	15 (62%)	23 (74%)	0.391
Hematogenous	32 (58%)	11 (46%)	21 (68%)	0.168
Peritoneum	26 (47%)	12 (50%)	14 (45%)	0.789
Organs with metastasis				
1	16 (29%)	10 (42%)	6 (19%)	0.083
≥ 2	39 (71%)	14 (58%)	25 (81%)	
Previous treatment regimens				
2	34 (62%)	17 (71%)	17 (55%)	0.459
3	16 (29)	5 (21%)	11 (35%)	
≥ 4	5 (9%)	2 (8%)	3 (10%)	
Previous therapies				
Pyrimidine analogs	53 (96%)	23 (96%)	30 (97%)	1
Platinum	47 (86%)	20 (83%)	27 (87%)	0.718
Taxane	52 (94%)	23 (96%)	29 (94%)	1
Ramucirumab	46 (84%)	20 (83%)	26 (84%)	0.471
Trastuzumab	11 (20%)	1 (4%)	10 (32%)	0.015
Irinotecan	14 (26%)	5 (21%)	9 (29%)	0.547
Treatment period before nivolumab (months)	14.9 (4.9–64.6)	14.5 (5.8–64.6)	15.0 (4.9–45.8)	0.819
Best overall response				
Responder	18 (33%)	12 (50%)	6 (19%)	0.022
Non-responder	37 (67%))	12 (50%)	25 (81%)	
Immune-related adverse events (≥grade 2)				
Positive	18 (33%)	9 (38%)	9 (29%)	0.57

### Association of clinical features with OS and PFS

Table 7 shows OS and PFS in patients treated with nivolumab. In the Cox proportional hazards regression model, the following variables were identified as being significantly associated with a poor outcome: ECOG PS ≥1 (*p* = 0.018), prior treatment with trastuzumab (*p* = 0.040), no irAEs (*p* = 0.017), no response to nivolumab (*p* < 0.001), and inability to receive subsequent chemotherapy (*p* < 0.001). Other than non-responsiveness to nivolumab, no variables were significantly associated with PFS.

**Table 7:** Overall and progression-free survival of patients treated by nivolumab using cox proportional hazard regression model

Variable	Overall survival			Progression-free survival		
	HR	95% C.I.	*p* value	HR	95% C.I.	*p* value
Sex						
male	1			1		
female	1.196	0.608–2.353	0.604	1.714	0.913–3.220	0.094
Age						
≤ 66	1			1		
> 66	1.005	0.565–1.786	0.987	0.8503	0.492–1.470	0.561
ECOG PS						
0	1			1		
≥ 1	3.472	1.236–9.753	0.018	1.49	0.698–3.182	0.303
Primary site						
Gastric	1			1		
Esophagogastric junction	0.8768	0.392–1.963	0.749	0.669	0.301–1.487	0.324
Disease status						
Metastatic	1			1		
Relapsed	1.241	0.321–4.798	0.755	0.903	0.4899.-1.665	0.744
Histological type						
Intestinal type	1			1		
Diffuse type	0.881	0.490–1.581	0.67	1.067	0.622–1.833	0.813
Organ with metastasis						
1	1			1		
≥ 2	1.656	0.858–3.196	0.133	1.699	0.932–3.097	0.084
Lymph node metastasis						
no	1			1		
yes	0.995	0.529–1.871	0.987	0.959	0.539–1.707	0.888
Hematogenous metastasis						
no	1			1		
yes	1.576	0.871–2.853	0.133	1.491	0.860–2.584	0.155
Peritoneal metastasis						
no	1			1		
yes	1.049	0.591–1.862	0.8701	0.969	0.566–1.659	0.909
Previous treatment regimens						
2	1			1		
≥ 3	1.325	0.742–2.367	0.342	1.103	0.636–1.911	0.728
HER-2 status						
no	1			1		
yes	1.923	0.985–3.754	0.055	1.7	0.886–3.262	0.111
Prior trastuzumab						
no	1			1		
yes	2.123	1.036–4.352	0.040	1.672	0.844–3.313	0.141
Prior ramucirumab						
no	1			1		
yes	1.082	0.504–2.319	0.841	1.265	0.616–2.596	0.522
Treatment period before nivolumab						
≤ 14.9	1			1		
> 14.9	0.924	0.521–1.639	0.787	0.889	0.520–1.519	0.666
Immune-related adverse events (≥grade 2)						
no	1			1		
yes	0.3174	0.124–0.815	0.017	0.615	0.298–1.271	0.19
Best overall response						
Non-responder	1			1		
Responder	0.14	0.065–0.302	< 0.001	0.036	0.011–0.113	< 0.001
Subsequent chemotherapy after nivolumab						
No	1			1		
Yes	0.31	0.169–0.567	< 0.001	0.596	0.346–1.025	0.061

## Discussion

This multicenter retrospective study reports the results of nivolumab monotherapy in patients treated according to the ATTRACTION-2 trial schedule [9] for metastatic and relapsed gastric or esophagogastric junction cancer who were refractory to or intolerant of at least two previous chemotherapy regimens. In this study, the objective response rate was 7% and the DCR was 36%. Median OS was 5.95 months and PFS was 1.6 months. OS and PFS were significantly better in the group that responded to nivolumab. Prognostic factors that predicted poor OS after initiation of nivolumab monotherapy included an ECOG PS ≥1, history of treatment with trastuzumab, no irAEs, lack of response to nivolumab, and inability to receive subsequent chemotherapy.

In the ATTRACTION-2 trial [9, 10], patients who received nivolumab had a median OS of 5.3 months (95% CI 4.60–6.37) and a 12-month OS rate of 26.2% (95% CI 20.7–32.0). Median OS after nivolumab monotherapy for advanced or recurrent gastric/esophagogastric junctional cancer in patients who had received two or more chemotherapy regimens was reported to be 4.3–7.6 months in several retrospective studies [16–20], which is similar to the median OS time of 5.95 months in this study. The ATTRACTION-2 trial demonstrated the efficacy of nivolumab in Asian patients with pretreated AGC. Similarly in Western patients with AGC, nivolumab has been shown to be feasible and effective [20]. However, the number of patients with gastric cancer who benefit from nivolumab is limited, and it is necessary to identify biomarkers that can predict the outcome of treatment with this agent. Several studies have identified prognostic biomarkers of the effect of nivolumab monotherapy [9, 16–24]. However, several reports suggest that patients with AGC and poor PS derive limited survival benefit from nivolumab [9, 17, 18]. As shown in several studies of pembrolizumab in patients who had previously been treated for AGC [25, 26], better ECOG PS was associated with a higher response rate and longer OS in those who were treated with an immune checkpoint inhibitor (ICI). Considering that nivolumab is an ICI that exerts an antitumor effect by activating tumor immunity, it is probable that the efficacy of nivolumab would be limited in patients with poor PS because of decreased immunity. For the same reason, the Glasgow prognostic score, neutrophil-lymphocyte ratio, prognostic nutrition index score, and skeletal muscle loss have been reported to affect the outcomes of treatment with nivolumab in patients with AGC [16, 19, 23]. The presence of a systemic inflammatory response and the associated poor nutritional status, indicating a low prognostic nutrition index score and high neutrophil-lymphocyte ratio, might adversely affect compliance with nivolumab for advanced gastric cancer [19]. Furthermore, patients with better ECOG PS at the start of nivolumab had a significantly higher rate of transition to subsequent chemotherapy after nivolumab, and this subsequent chemotherapy significantly contributed to OS improvement. Arigami et al. [27] report that nivolumab exposure may enhance subsequent chemosensitivity in patients with AGC, and our findings may support it.

In this study, 10 patients (17%) experienced grade 2 or higher irAEs, and OS in these patients was significantly higher than that in those without irAEs. Development of irAEs is reportedly associated with better survival outcomes in various types of cancer, including AGC [19, 24, 28–30]. By inhibiting PD-1 on T-cells, nivolumab reactivates suppressed T-cells and has antitumor effects. Given that irAEs are manifestations of the immune response through T-cell activation, they are likely to be related to the antitumor effect of nivolumab. Furthermore, T-cells enhance the effect of treatment with the PD-1 antibody, which may in turn induce autoantibodies via B-cells, thereby promoting the development of irAEs [21, 29]. Therefore, manifestation of irAEs might be a useful predictor of the response to nivolumab in patients with AGC.

Previous treatment with trastuzumab was associated with poor OS in patients with AGC who were treated with nivolumab, which is in contrast with the findings of the ATTRACTION-2 study [31]. Although the relationship between prior trastuzumab use and the therapeutic effect of nivolumab has not been clarified, the following mechanism has been implicated. Trastuzumab has been reported to induce rapid increases in localization of phosphatase and tensin homolog (PTEN) to the membrane and phosphatase activity by reducing PTEN tyrosine phosphorylation via Src inhibition [32]. That is, trastuzumab has an antitumor effect via activation of PTEN, and loss of PTEN is predicted to be involved in resistance to trastuzumab. Furthermore, previous studies have shown that loss of PTEN contributes to resistance to T-cell-mediated immunotherapy [33, 34]. From the perspective of loss of PTEN, nivolumab may be less effective in patients with AGC who are resistant to trastuzumab, and further molecular biological studies may be needed.

Until now, nivolumab has been limited to third line treatment in the indications of gastric cancer and esophagogastric junction cancer after failure of two or more alternative treatment regimens, but it is expected to be effective in the first line, second line, and adjuvant therapy in the future [35–38]. In the results of the randomized open-label Phase III CheckMate 649 study, the efficacy of nivolumab as first-line treatment in combination with chemotherapy have been reported [35]. In the CheckMate 577 study, it has been reported that disease-free survival was significantly longer in patients with resected esophageal or gastroesophageal junction cancer who received nivolumab as adjuvant therapy after neoadjuvant chemoradiotherapy than in those who received placebo as adjuvant therapy [38].

This study has some limitations. First, it had a single-arm, retrospective, non-randomized observational design and included a relatively small number of patients. Therefore, although the study was conducted at multiple centers, the possibility of selection bias cannot be excluded. Second, a multivariate analysis could not be conducted because of the relatively small cohort size. Third, poor prognostic factors were identified based on clinical data with no molecular biological analysis, especially regarding the correlation between prior trastuzumab use and nivolumab refractory. Further studies are required in the future.

## Conclusions

In conclusion, this multicenter retrospective study identified that an ECOG PS of 0, no prior treatment with trastuzumab, presence of irAEs, response to nivolumab, and ability to administer chemotherapy subsequent to nivolumab were potential prognostic markers of prolonged OS after initiation of nivolumab in patients with AGC. Our study findings suggest that nivolumab should not recommended in patients with AGC who have poor PS and those who have previously been treated with trastuzumab. Further molecular biological studies are needed, in particular to identify the mechanism of intolerance to nivolumab in patients with AGC that is resistant to trastuzumab.

## Data Availability

The datasets generated during and/or analyzed during the current study are available from the corresponding author on reasonable request.

## Abbreviations

AGC: Advanced or recurrent gastric cancer
HER2: Human epidermal growth factor receptor 2
PD-1: Programmed cell death-1
OS: Overall survival
HR: Hazard ratio
ECOG PS: Eastern cooperative oncology group performance status
CR: Complete remission
PR: Partial response
SD: Stable disease
PD: Progressive disease
RECIST: Response evaluation criteria in solid tumors
AEs: Adverse events
irAEs: Immune-related adverse events
PFS: Progression-free survival
CIs: Confidence intervals
DCR: Disease control rate
TRAEs: Treatment-related adverse events
ICI: Immune checkpoint inhibitor
PTEN: Phosphatase and tensin homolog
